# The Pharmaceutical Device Prisma^®^ Skin Promotes in Vitro Angiogenesis through Endothelial to Mesenchymal Transition during Skin Wound Healing

**DOI:** 10.3390/ijms18081614

**Published:** 2017-07-25

**Authors:** Raffaella Belvedere, Valentina Bizzarro, Luca Parente, Francesco Petrella, Antonello Petrella

**Affiliations:** 1 Department of Pharmacy, University of Salerno, via Giovanni Paolo II 132, 84084 Fisciano (Salerno), Italy; rbelvedere@unisa.it (R.B.); vbizzarro@unisa.it (V.B.); lparente@unisa.it (L.P.); 2 Primary Care—Wound Care Service, Health Local Agency Naples 3 South, Via Libertà 42, 80055 Portici (Napoli), Italy; dottpetrella@gmail.com

**Keywords:** mesoglycan, Prisma^®^ Skin, glycosaminoglycans, angiogenesis, skin wound healing

## Abstract

Glycosaminoglycans are polysaccharides of the extracellular matrix supporting skin wound closure. Mesoglycan is a mixture of glycosaminoglycans such as chondroitin-, dermatan-, heparan-sulfate and heparin and is the main component of Prisma^®^ Skin, a pharmaceutical device developed by Mediolanum Farmaceutici S.p.a. Here, we show the in vitro effects of this device in the new vessels formation by endothelial cells, since angiogenesis represents a key moment in wound healing. We found a strong increase of migration and invasion rates of these cells treated with mesoglycan and Prisma^®^ Skin which mediate the activation of the pathway triggered by CD44 receptor. Furthermore, endothelial cells form longer capillary-like structures with a great number of branches, in the presence of the same treatments. Thus, the device, thanks to the mesoglycan, leads the cells to the Endothelial-to-Mesenchymal Transition, suggesting the switch to a fibroblast-like phenotype, as shown by immunofluorescence assays. Finally, we found that mesoglycan and Prisma^®^ Skin inhibit inflammatory reactions such as nitric oxide secretion and NF-κB nuclear translocation in endothelial cells and Tumor Necrosis Factor-α production by macrophages. In conclusion, based on our data, we suggest that Prisma^®^ Skin may be able to accelerate angiogenesis in skin wound healing, and regulate inflammation avoiding chronic, thus pathological, responses.

## 1. Introduction

The healing of adult skin wound is a complex process requiring the collaboration of many different cell populations which have specific roles during the three classical stages: inflammation, new tissue generation and remodeling [1,2]. While the inflammation occurs immediately after tissue damage, new tissue formation takes place 2–10 days after injury and is characterized by proliferation and migration of different cell types. Firstly, keratinocytes migrate over the injured dermis, then new blood vessels are needed to allow fibroblasts and leukocytes to replace the fibrin matrix with granulation tissue, which forms a new substrate for keratinocyte migration at later stages of the repair process [3]. Thus, a critical component of the wound healing is represented by angiogenesis. The latter is an event requiring a dynamic temporally and spatially regulated interaction between endothelial cells, angiogenesis factors, and surrounding extracellular matrix (ECM) proteins [4]. One of the several processes playing a crucial role in stabilizing the neovasculature during angiogenesis is the Endothelial-to-Mesenchymal Transition (EndMT), a specific form of epithelial–mesenchymal transition (EMT). During EndMT, resident endothelial cells delaminate from an organized cell layer and acquire a mesenchymal phenotype characterized by loss of cell–cell junctions, loss of endothelial markers, gain of mesenchymal markers, and acquisition of invasive and migratory properties. EndMT-derived cells are believed to function as fibroblasts-like ones in damaged tissue actively contributing to tissue remodeling [5,6].

The formation of new blood vessels is indispensable for successful wound healing [7]. To allow a precise control of blood vessel regeneration during repair, endothelial cells also have to interact with a soluble angiogenic growth factors and ECM components. Among these molecules, glycosaminoglycans (GAGs), unbranched polysaccharides with repetitive disaccharide units, define skin volume and elasticity and are reported to be able to improve skin wound healing, even though they represent only the 0.1–0.3% of the total skin weight. Chondroitin sulfate (CS), dermatan sulfate (DS), keratan sulfate (KS), heparan sulfate (HS) and heparin (HEP) are a class of GAGs distributed on the surface of all cells and in ECM [8]. The biological properties of these biomacromolecules are strongly influenced by the degree of sulfation, and the sulfate group distribution along the polymer renders certain GAGs interesting pharmacological molecules mainly in tissue repair [9]. When they are linked to protein core, they form proteoglycans (PGs) and become able to play a scaffold role and bind a variety of ligands functionally important in morphogenesis. During the proliferation phase of wound healing, the de novo synthesis of GAGs, including hyaluronic acid (HA), increases temporarily to modulate fibroblasts physiology, and supports keratinocyte proliferation and migration [10,11]. Nowadays, the role of GAGs in the angiogenesis represents an interesting research object as PGs containing HS side chains, e.g., perlecan and collagen XVIII, are important to sequester and store growth factor such as Fibroblasts Growth Factor (FGF)-2 [12]. Their importance has been further confirmed in the study on mice by Zhou et al. showing that the number of blood vessels is reduced when exon region containing the GAG chain attachment sites was removed [13]. 

Furthermore, miming the action of GAGs, it is possible to stimulate the inflammation resolution and the increase of the neovascularization [14]. Indeed, in the inflammatory phase, GAGs direct the migration of neutrophils and macrophages towards the wounded tissue where they are also known to act as immunosuppressants blocking NF-κB signaling pathway and downregulating the expression of several pro-inflammatory cytokines [11]. 

As natural GAG preparation extracted from porcine intestinal mucosa, mesoglycan is now used for the treatment of vascular disease with an associated thrombotic risk such as deep venous thrombosis and chronic venous insufficiency. It is able to inhibit platelet adhesion and smooth muscle cells proliferation and to stimulate lipoprotein lipase enzyme. To these activities, mesoglycan also associates anti-thrombotic and profibrinolytic effects, due to the presence of HS and DS, fundamental constituents of the vessel wall. In addition, it is able to restore the physiological properties of selective barriers of capillary endothelium with an anti-edema activity [15,16,17,18]. Mesoglycan is composed of HS (47.5%), DS (35.5%), slow-moving HEP (8.5%) and CS (8.5%) and represent the main component of Prisma^®^ Skin, a pharmaceutical device including also hyaluronic acid in an alginate water-soluble dressing on inert polyethylene terephthalate (PET) support material. We previously showed the use of mesoglycan and, particularly, Prisma^®^ Skin, in skin wound healing is able to enhance re-epithelialization and granulation processes in vitro [19]. We tested this device on adult dermal fibroblasts and epidermal keratinocytes, two cell populations whose activation supports a good resolution of skin injury, finding a strong increase of cell migration and invasion due to a notable cytoskeleton reorganization [19]. 

In this work, we continued the study of the effects of Prisma^®^ Skin focusing on human endothelial cells and macrophages to investigate new blood vessel formation and inflammatory response, key phases leading to the completion of epithelialization upon the remodeling phase in skin wound healing.

## 2. Results

### 2.1. HUVEC (Human Umbilical Vein Endothelial Cells) Cells Viability Was Not Affected by Mesoglycan and Prisma^®^ Skin

**Figure 1:**
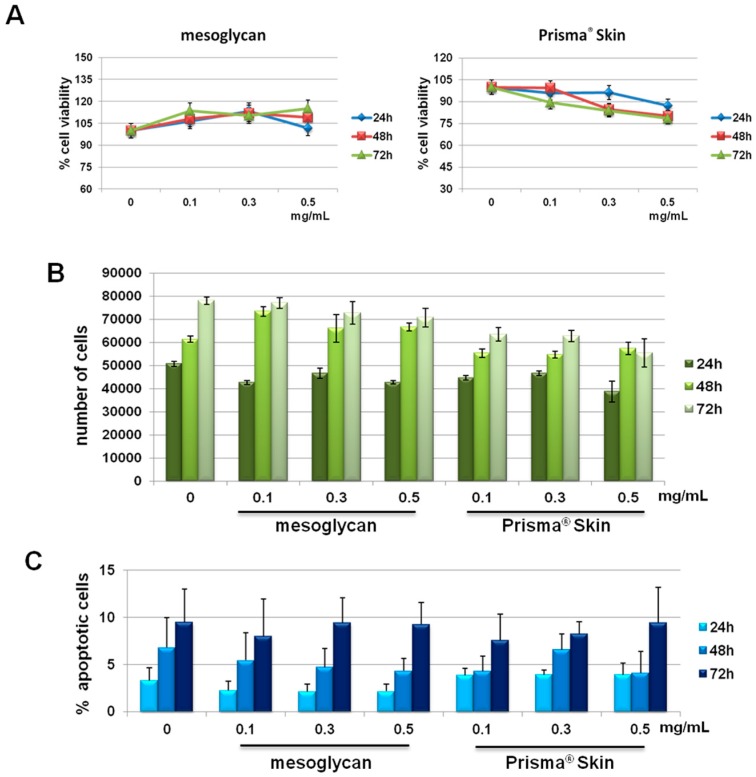
(**A**) MTT (3-(4,5-Dimethylthiazol-2-yl)-2,5-Diphenyltetrazolium Bromide) assay on HUVEC cells evaluating cell viability. Absorbance relative to controls was used to determine the percentage of cells treated with sodium mesoglycan and Prisma^®^ Skin 0.1, 0.3 and 0.5 mg/mL at 24, 48 and 72 h. The values reported in the graphs are the mean ± S.E.M. from five independent experiments performed in triplicates, results appeared not significant (ns, *p* > 0.05); (**B**) Hemocytometer counting of HUVEC cells treated or not with mesoglycan and Prisma^®^ Skin. The data are representative of five experiments with similar results; (**C**) Analysis of apoptotic cells by cytofluorimetric assay of the effect of 0.1, 0.3 and 0.5 mg/mL of mesoglycan and Prisma^®^ Skin at 24, 48 and 72 h. The data are the mean of five experiments with similar results (ns, *p* > 0.05, based on Student’s t-test, assuming a two-tailed distribution and unequal variance).

We previously reported the effects of sodium mesoglycan and Prisma^®^ Skin on human keratinocytes and fibroblasts [19]. We followed the investigation on HUVEC cells. First, we focused on the evaluation of a potential cytotoxicity of mesoglycan and Prisma^®^ Skin 0.1, 0.3, 0.5 mg/mL. As reported in Figure 1A, MTT assay did not show any significant modulation of cell viability at 24, 48 and 72 h from treatments. We confirmed the lack of toxicity by hemocytometer counting at the same experimental points (Figure 1B). Finally, we further performed the analysis of apoptosis by staining with PI, as reported in Materials and Methods Section. We did not find any significant apoptotic signal in presence of mesoglycan and Prisma^®^ Skin 0.1, 0.3, 0.5 mg/mL at 24, 48 and 72 h of treatment if compared with untreated cells (Figure 1C).

### 2.2. Mesoglycan and Prisma^®^ Skin Positively Affected the HUVEC Migration and Invasion Rate

**Figure 2:**
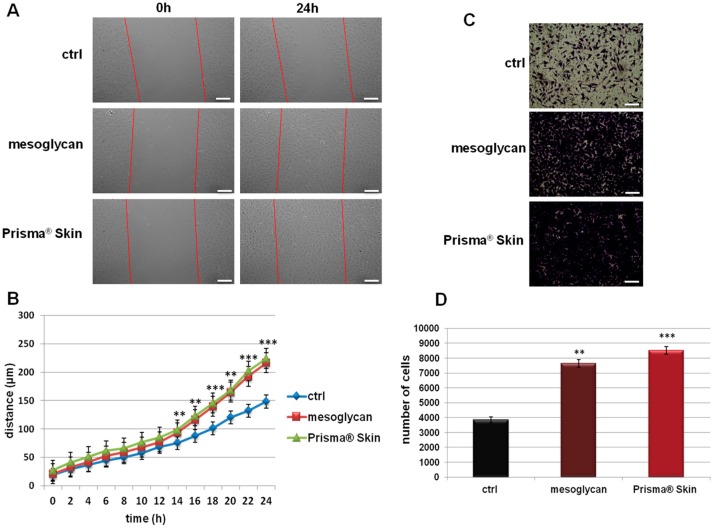
(**A**) Representative images of Wound Healing assay on HUVEC cells treated or not with sodium mesoglycan and Prisma^®^ Skin 0.3 mg/mL. Bar: 100µm; (**B**) Results of Wound Healing assay analysis. The migration rate of individual cells was determined by measuring the distances covered from the initial time to the selected time-points (bar of distance tool, Leica ASF software). The data represent a mean of three independent experiments ± SEM, their statistical significance was evaluated using Student’s *t*-test, assuming a two-tailed distribution and unequal variance. ** *p* < 0.01; *** *p* < 0.001; (**C**) Representative images of analyzed fields of invasion assay; (**D**) Analysis of invasion speed of HUVEC cells with mesoglycan and Prisma^®^ Skin. Data represent the mean cell counts of 12 separate fields per well ± SEM of five experiments with similar results. Bar: 50µm. ** *p* < 0.01; *** *p* < 0.001.

Because endothelial cell migration and invasion play an essential role during the angiogenesis, we investigated how sodium mesoglycan and the device Prisma^®^ Skin could influence these processes. As shown in Figure 2A,B, the migration rate of HUVEC cells is strongly enhanced by mesoglycan and by Prisma^®^ Skin at 0.3 mg/mL. Particularly, at 24 h, mesoglycan and Prisma^®^ Skin treated cells moved towards wound closure 68% and 76% more than control cells, respectively. To investigate cell invasiveness ability, we performed functional assays plating HUVEC on the upper chamber of trans-wells and administrating mesoglycan and Prisma^®^ Skin in the lower chamber for 24 h. In this way, we found that the invasive rate increased by 49% in presence of mesoglycan and by 55% with Prisma^®^ Skin (Figure 2C,D).

### 2.3. CD44 Pathway Was Influenced by Mesoglycan and Prisma^®^ Skin

**Figure 3:**
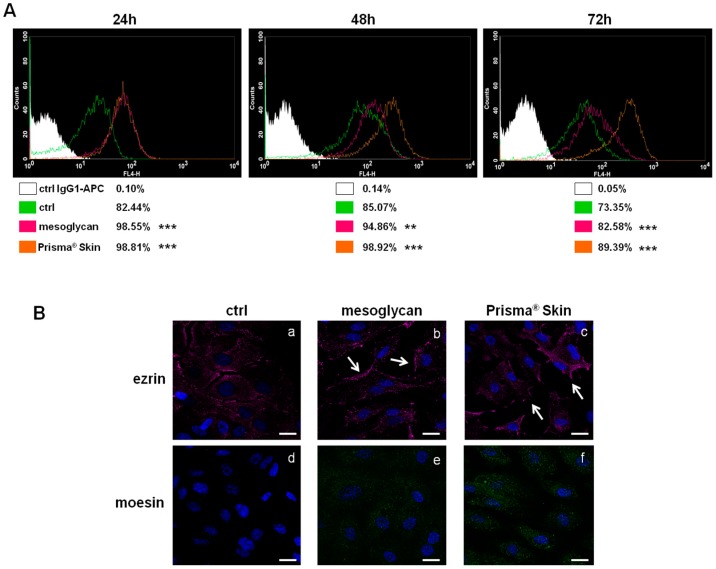
(**A**) Cell surface expression of CD44 was analyzed by flow cytometry at 24, 48 and 72 h from the administration of mesoglycan and Prisma^®^ Skin. The white areas in the plots are relative to human IgG1; CD44 signals are shown in green for ctrl HUVEC, in purple for HUVEC in presence of mesoglycan and in ocher for cells with Prisma^®^ Skin. The results are representative of the mean ± SEM of three analyzed experiments. ** *p* < 0.01; *** *p* < 0.001; (**B**) Immunofluorescence analysis to detect ezrin (**a**–**c**, white arrows for membrane localization) and moesin (**d**–**f**) treated with mesoglycan and Prisma^®^ Skin for 48 h. Nuclei were stained with DAPI (4′,6-Diamidino-2-Phenylindole). Magnification 63× 1.4 NA (Numerical Aperture). Bar = 10 µm.

The surface receptor CD44, through interactions with its ligands, is highly implicated in the formation of new blood vessels [20,21]. We investigated the expression of CD44 on HUVEC cells based on the huge amount of studies that showed its function on endothelial cells [21,22]. As shown in Figure 3A, CD44 expression significantly increased at 24, 48 and 72 h with mesoglycan and with Prisma^®^ Skin, compared to untreated cells. This receptor, particularly on endothelial cells, activates intracellular signals required in reorganization of the cell cytoskeleton during cell directional movement. Indeed, the cytoplasmic domain of CD44 recruits ERM proteins (ezrin, radixin, and moesin) that bind the actin cytoskeleton and promote activation of Ras once activated by PKC phosphorylation [23]. To verify the involvement of ERM complex, we performed an immunofluorescence assay on HUVEC cells showing that ezrin protein notably translocated to plasma membrane at 48 h of treatment with sodium mesoglycan and Prisma^®^ Skin (Figure 3(Ba–c), white arrows). Ezrin relocalization was also associated with the increase of moesin expression (Figure 3(Bd–f)).

### 2.4. Mesoglycan and Prisma^®^ Skin Induced HUVEC Cells to Regulate Tube Formation

**Figure 4:**
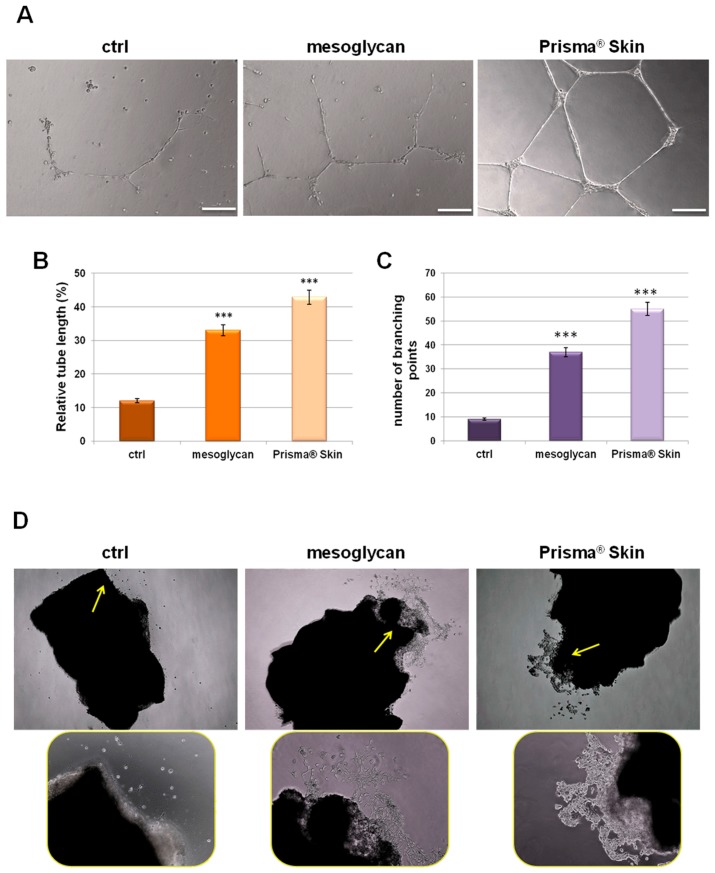
(**A**) Representative images of tube formation by HUVEC cells seeded for 12 h on matrigel: EBM-2 1:1 and treated or not with sodium mesoglycan and Prisma^®^ Skin 0.3 mg/mL. Bar: 100µm. Analysis of: (**B**) tube length; and (**C**) number of branches calculated by ImageJ (Angiogenesis Analyzer tool) software. Data represent the mean of three independent experiments ± SEM with similar results. *** *p* < 0.001; (**D**) Representative bright field images of mouse aortic sections captured at EVOS light microscope 4× (rectangular pictures) and 10× (rounded pictures are enlargements of areas indicated by yellow arrows).

Our characterization of angiogenesis process started with the study of endothelial cell migration and invasion promoted by mesoglycan and particularly by Prisma^®^ Skin 0.3 mg/mL. We continued this work evaluating the tubulogenesis since the endothelial cells form capillary-like structures in response to signals found in conditioned media. We found that sodium mesoglycan and Prisma^®^ Skin functioned as pro-angiogenic signals enhancing the formation of these structures after 12 h of treatment with respect to control (Figure 4A). The analysis of length and the counting of the branches of tubes generated in presence of mesoglycan and Prisma^®^ Skin (Figure 4B,C) revealed that treatments significantly improved the build of these new formed structures if compared to ones generated by untreated HUVEC cells. Additionally, we provided ex vivo mouse aortic section sprouting assays supporting our analysis of endothelial cell migration and allowing us to directly compare the pro-angiogenic effects of mesoglycan and Prisma^®^ Skin. We could identify cells that moved from aortic tissue section with an increasing rate in the case of treatment with mesoglycan and Prisma^®^ Skin 0.3 mg/mL if compared to untreated sections (Figure 4D, yellow arrows).

### 2.5. Endothelial-to-Mesenchymal Transition May Be Initiated by Mesoglycan and Prisma^®^ Skin

**Figure 5:**
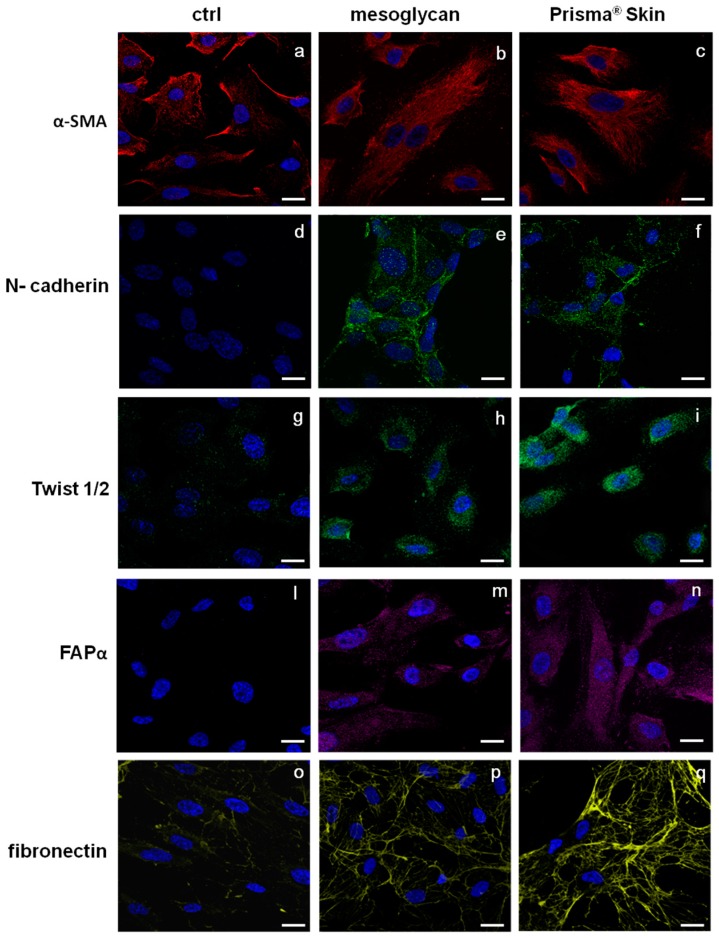
Immunofluorescence analysis to detect: α-SMA (**a–c**); N-cadherin (**d**–**f**), Twist 1/2 (**g–i**); FAPα (**l**–**n**); and fibronectin (**o**–**q**) treated with mesoglycan and Prisma^®^ Skin for 48 h. Nuclei were stained with DAPI. Magnification 63× 1.4 NA. Bar = 10 µm. All the results are representative of three experiments with similar results.

The endothelium may undergo EndMT in direct response to tissue injury and transitioning endothelial cells acquire a migratory phenotype, invade under the vascular basement membrane, and begin to express mesenchymal markers [24,25]. EndMT may play an important role in stabilizing the neovasculature during vasculogenesis and angiogenesis. Therefore, we investigated the most important protein markers whose modulated expression could contribute to the stabilization of an EndMT-derived subpopulation. Figure 5a–c reports the expression of α-smooth muscle actin (SMA), a well established marker for mesenchymal phenotype and for early EndMT [5]. α-SMA is expressed by HUVEC cells but not structured as a typical cytoskeleton protein. On the contrary, in the case of HUVEC treatment for 48 h with mesoglycan and Prisma^®^ Skin, α-SMA acquired an organized intracellular distribution (Figure 5b,c) as it appears in activated fibroblast deriving from switched endothelial cells [26]. Other simultaneous distinct changes involved the acquisition of *N*-cadherin, typical marker of cells which have lost cell-cell junctions and increased motility [27], as it appears in HUVEC cells treated with mesoglycan and Prisma^®^ Skin (Figure 5d–f). 

A number of autocrine or paracrine signaling molecules can induce EndMT as transforming growth factor-β (TGF-β) superfamily, Bone Morphogenetic Proteins (BMPs), Wnt/β-catenin, Notch and various receptor tyrosine kinases [28]. All these factors induce the activation of several transcriptional factors that cause the repression of endothelial genes and/or expression of mesenchymal ones. Among them, we found that HUVEC cells showed a significant nuclear translocation of Twist 1/2 after 48 h of treatments with mesoglycan and Prisma^®^ Skin (Figure 5g–i).

To further confirm the switch to fibroblast-like characteristics, we stained treated or not HUVEC cells for fibroblast activated protein (FAP)-α and we found that only in presence of mesoglycan and Prisma^®^ Skin this protein became notably expressed (Figure 5l–n). 

Moreover, we evaluated also changes in the expression of fibronectin protein, as it is able to promote cell migration through cytoskeletal reorganization. Then, confirming the highly invasive and migratory ability of these newly formed mesenchymal cells, also fibronectin expression strongly increased in presence of mesoglycan and Prisma^®^ Skin (Figure 5o–q).

### 2.6. Mesoglycan and Prisma^®^ Skin Showed Anti-Inflammatory Effects on HUVEC and SC Cells

**Figure 6:**
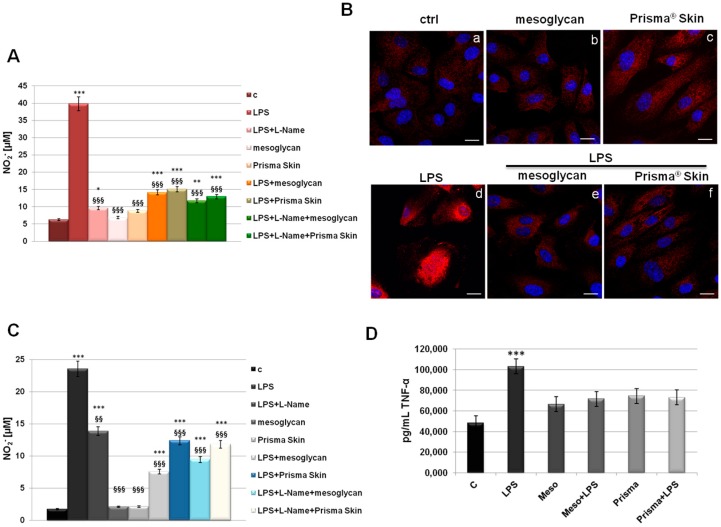
(**A**) NO (nitric oxide) release detected as [NO_2_^−^] µM by Griess reaction in presence of LPS (10 µg/mL) for 24 h, with or without L-Name (100 µM), mesoglycan and Prisma^®^ Skin 0.3 mg/mL; (**B**) HUVEC cells were treated with LPS (10 µg/mL) for 24 h and co-exposed or not with sodium mesoglycan and Prisma^®^ Skin 0.3 mg/mL and nuclear translocation of NF-κB p65 subunit was detected using immunofluorescence assay at confocal microscopy. Nuclei were stained with DAPI. Magnification 63× 1.4 NA. Bar = 10 µm. All the results are representative of 3 experiments with similar results; (**C**) NO release detected on SC cells in presence of LPS (10 µg/mL) for 24 h, with or without L-Name (100 µM), mesoglycan and Prisma^®^ Skin 0.3 mg/mL. As for HUVEC cells, data derive from the mean of three independent experiments showing similar results and have been analyzed using Student’s *t*-test, assuming a two-tailed distribution and unequal variance. * *p* < 0.05; ** *p* < 0.01; *** *p* < 0.001 for all treatments vs. untreated cells. ^§§^ *p* < 0.01; ^§§§^ *p* < 0.001 for all treatments vs. LPS-treated cells; (**D**) TNF-α production was measured in the supernatants of SC cells by ELISA kit. Cells were treated with LPS (10 µg/mL) alone or in combination with mesoglycan and Prisma^®^ Skin 0.3 mg/mL for 24 h. Data represent the mean of three independent experiments with similar results and analyzed by Student’s *t*-test, assuming a two-tailed distribution and unequal variance. *** *p* < 0.001.

The inflammatory reactions represent one of the first steps evolving at the site of injury to provide key functions needed for the influx of connective tissue during skin wound healing. This efficient process is lost in pathological conditions developing in a status of chronic inflammation [29]. To assess if mesoglycan and Prisma^®^ Skin contribute to the modulation of the inflammatory response during skin wound healing, we measured NO_2_^−^ release, as a stable end-product of nitric oxide (NO), in the medium of HUVEC cells previously treated by LPS (10 µg/mL) alone and in combination with our reference substances. We further used L-Name as technical control since it represents a direct inhibitor of NO synthesis, as confirmed in our data. In Figure 6A, we showed a marked increase of NO_2_^−^ production at 24 h after LPS administration, on the other hand, a significant reduction of NO synthesis appeared when LPS have been administrated together with mesoglycan and Prisma^®^ Skin. Notably, these compounds did not show any relevant effect on NO production when used alone. Another important effect induced by LPS inflammatory stimulus that we considered is the activation by phosphorylation of the free NF-κB dimers which translocate into the nucleus and bind to specific sequences to regulate downstream genes expression [30]. Thus, we labelled p65 with red fluorescence to track the influence of mesoglycan and Prisma^®^ Skin added to HUVEC cells with or without LPS (10 µg/mL) on NF-κB translocation. As shown in Figure 6B, nuclear p65 was increased after 24 h by LPS, on the contrary this process was strongly reduced by mesoglycan and Prisma^®^ Skin 0.3 mg/mL alone and in presence of LPS. 

Furthermore, we focused on the macrophage implication in the inflammatory response. In fact, macrophages infiltrate the wound massively two days post injury exacerbating an intense phagocytic activity. Schematically, macrophages activated by microbial agents and cytokines such as Interferon γ (IFNγ) produce an important level of NO and pro inflammatory cytokines such as TNF-α, IL-1β, IL-6, or IL-12 [31,32]. As revealed for HUVEC, on SC cells LPS induced a strong production of NO, expressed as NO_2_^−^ concentration in cell medium, and this reaction is notably reverted in presence of mesoglycan and Prisma^®^ Skin 0.3 mg/mL (Figure 6C). Additionally, we performed an ELISA assay for TNF-α on the supernatants of macrophages treated with LPS in combination or not with sodium mesoglycan and Prisma^®^ Skin. Similarly, we found that our compounds are able to inhibit the pro-inflammatory effects of LPS such as TNF-α secretion. Moreover, mesoglycan and Prisma^®^ Skin alone did not affect the production of this cytokine (Figure 6D). 

## 3. Discussion

The non-healing or chronic skin wound can be due to several mechanisms including a reduced bioavailability of growth factors, abnormal production or modification of matrix proteins, diminished proliferative capacity of resident cells, and insufficient wound perfusion [7]. The correct sequence of these mechanisms provides chemotactic cues to recruit circulating inflammatory cells to the wound site, initiate the re-epithelialization and connective tissue contraction, and stimulate the characteristic wound angiogenic response [1].

Many clinical studies reported the beneficial effect of mesoglycan in the treatment of vascular diseases thanks to its anti-thrombotic and profibrinolytic activities in an acute phase as well as in prolonged therapy [33,34]. Moreover, our previous works showed that Prisma^®^ Skin is able to contribute to the formation of granulation tissue enhancing the dermal fibroblasts proliferation, migration and invasion and the re-epithelialization by keratinocytes thanks to the presence of mesoglycan, as its main component. [19,35]. In this study, we investigated the in vitro effects of this device on the formation of new blood vessels and the inflammatory response since these processes represent a paradigmatic model to investigate the molecular mechanisms involved in the skin remodeling. 

First, it was verified that sodium mesoglycan and Prisma^®^ Skin were not cytotoxic on human endothelial cells and macrophages (data not shown), HUVEC and SC cell lines, respectively. Then, we proceeded analyzing the migration and invasion rate of HUVEC cells which are strongly enhanced by mesoglycan and particularly by Prisma^®^ Skin, probably thanks to the additional presence of HA and alginates in the whole device. These results are consistent with the current approaches for therapeutic tissue regeneration based on the interactions between the growth factors, such as VEGF (Vascular Endothelial Growth Factor), and the molecules of the ECM, such as HA and GAGs [36]. Furthermore, it is reported that endothelial cells and fibroblasts form a functional unit essential for efficient blood vessel growth during tissue granulation. Fibroblasts deposit a complex provisional wound matrix consisting of GAGs, PGs, collagen III and other proteins of ECM which promote endothelial migration culminating in tube formation and vessel growth [2]. Additionally, endothelial cells detach from the basement membrane and remove some of the major components of ECM to invade it [37]. 

One of the most characterized candidates as surface receptor promoting cell locomotion is CD44, well-known to bind HA fragments and able to induce endothelial cell proliferation and migration [38]. CD44 is expressed on epithelial cells, bone marrow-derived cells such as recruited leukocytes, tissue macrophages, and circulating endothelial progenitor cells, as multiple isoforms containing alternatively spliced exons [39]. The increased expression of CD44 on HUVEC cells following the administration of mesoglycan and Prisma^®^ Skin confirmed the involvement of the mechanism triggered by this receptor on the endothelium as previously revealed on keratinocytes [19]. Furthermore, CD44 acts in the stabilization and maturation of newly formed vessels mediating the early stages of the cell-substrate interaction, promoting the remodeling of the endothelial pericellular matrix. This leads to the attachment of heparin-binding angiogenic factors such as VEGF and basic Fibroblast Growth Factor (bFGF) [40]. This last occurrence is supported by the translocation to the plasma membrane of ezrin and the increase of moesin expression, two key events which led us to consider the involvement of the pathway CD44-ERM complex in the control of the cytoskeletal dynamism during cell invasion [41] and angiogenesis. Therefore, we evaluated the in vitro pro-angiogenic effects of mesoglycan and Prisma^®^ Skin highlighting the increased number of branches and the length of new formed vessels on coating of matrigel and the amount of cells spread from mice aortic fragments. To explain this strong angiogenic response, we focused on the EndMT as a process that may induce endothelial cells to acquire a variety of different mesenchymal features including secretion of ECM proteins such as fibronectin and collagen [25]. Beyond the increase of CD44 expression, we investigated the presence of other markers proving the induction of the EndMT. Whenever cells had been treated with mesoglycan and Prisma^®^ Skin, we found well organized filaments of α-SMA. This protein is expressed in HUVEC cells [42] and by endothelial cells fusiform in shape [43], suggesting an evolving early time point of EndMT. Moreover, the transformation of endothelial cells in the mesenchymal ones is further confirmed by the de novo expression of N-cadherin by HUVEC cells in presence of mesoglycan and Prisma^®^ Skin. N-cadherin is a membrane protein typically expressed by cell with an highly invasive and poorly polarized phenotype [44]. Its function in endothelium remains largely unknown; few studies reported an effect of soluble N-cadherin on migration and angiogenesis through the FGF-receptor, acting in opposition of VE-cadherin in vascular morphogenesis [45,46]. Generally, the reprogramming of gene expression in EndMT is mediated by key transcription factors, as Snail, Slug, ZEB-1, SIP-1, LEF-1, and Twist 1/2 which are responsible for the switch of endothelial cells to other cell types like fibroblasts or myofibroblasts. This feature is supported by the appearance of FAP-α, induced by mesoglycan and better by Prisma^®^ Skin. FAP-α is a serine proteinase and is considered the marker of reactive stromal fibroblasts surrounding the newly formed blood vessels in granulation tissue of healing wounds [47,48]. Finally, the increase of the expression of fibronectin suggests the replacement of the basal lamina by new matrix molecules during EMT/EndMT after its degradation by matrix metalloproteinases [49]. Indeed, endothelial cells are able to attach to ECM molecules such as fibrinogen, fibronectin, vitronectin, and von Willebrand factor through αvβ3 integrin, their specific receptor [4]. 

Therefore, EndMT should provide a natural and effective method for building new connective tissues from blood vessels. On the other hand, cell and tissue movements necessary for repair are strongly affected by the inflammatory response. Experimental studies established the notion that inflammation is essential for the cutaneous homeostasis following injury, now this notion has been overturned considering the chronic inflammation as a hallmark of the non-healing wound [50]. The anti-inflammatory effects of Prisma^®^ Skin and mesoglycan on endothelial cells and macrophages indicates a potent role of these agents in the regulation of the excessive and unbalanced inflammation characterizing the chronic wound. Accordingly, the device functions favoring skin wound healing closure also through the neutralization of NO synthesis and of NF-κB activation.

It is known that endothelial cells interact with both stromal and inflammatory cells, such as macrophages, in the microenvironment. The macrophages are activated by recognition of pathogen-associated molecular patterns, such as bacterial LPS and peptidoglycan, or damage-associated molecular patterns, such as released intracellular proteins and nucleic acids. Particularly, these cells are able to influence angiogenesis: they release chemotactic factors to attract fibroblasts to the wound area, and produce increased quantities of collagen leading to the granuloma. Besides resident macrophages, the major portion of these ones at the wound site is recruited from the blood [51]. Prisma^®^ Skin and sodium mesoglycan showed a strong reduction of macrophage activation, in term of TNF-α production and NO secretion, probably enhancing the switch to an anti-inflammatory phenotype as promoter of angiogenesis and, generally, wound healing closure [52]. 

## 4. Materials and Methods

### 4.1. Cell Cultures

HUVEC cell line was purchased from American Type Culture Collection (ATCC, Manassas, VA, USA) (ATCC® PCS-100-010™) and was maintained in endothelial growth medium (EGM-2) medium contains EBM-2 medium (serum free, growth-factor free), supplemented with 2% fetal bovine serum (FBS), human fibroblast growth factor-B (hFGF-B), human epidermal growth factor (hEGF), human vascular endothelial cell growth factor (hVEGF), long R insulin-like growth factor-1 (R3-IGF-1), ascorbic acid, hydrocortisone, and heparin (Lonza). Cells cultured until passage 10. SC cells (human monocytes/macrophages) were purchased from ATCC (ATCC^®^ CRL-9855™) and cultured in Iscove’s modified Dulbecco’s medium (Thermo Fisher Scientific, Waltham, MA, USA) with 4 mM l-glutamine and supplemented with 0.05 mM 2-mercaptoethanol, 0.1 mM hypoxanthine and 0.016 mM thymidine and 10% FBS (Euroclone). Cells were stained at 37 °C in 5% CO_2_—95% air humidified atmosphere. 

### 4.2. Preparation and Seeding of Mesoglycan and Prisma^®^ Skin

The compositions of powder of sodium mesoglycan and Prisma^®^ Skin medical device were described in [19] and were provided by Mediolanum Farmaceutici S.p.a. (Milan, Italy). Sodium mesoglycan was dissolved in cell medium at an initial concentration of 1 mg/mL. The sheets of Prisma^®^ Skin were treated as reported in [19]. To properly administrate the correspondent amount of mesoglycan, each sheet was dissolved in the medium to gain an initial concentration of 1 mg/mL of sodium mesoglycan. 

### 4.3. MTT Assay

After treatment with mesoglycan and Prisma^®^ Skin at 0.1, 0.3, 0.5 mg/mL HUVEC cells were harvested at the indicated times (24, 48 and 72 h) and cell viability was calculated as previously described [53]. The viability of cells in response to treatments with tested compounds was calculated as: % viable cells = (OD (550–690 nm) mesoglycan or Prisma^®^ Skin/OD (550–690 nm) negative control) × 100. The optical density (OD) of each well was measured with a spectrophotometer (Titertek, Multiskan MCC/340, Pforzheim, Germany) equipped with a 620 nm filter. 

### 4.4. Hemocytometer-Cell Counting

First, 2 × 10^4^ HUVEC cells were seeded on a 12 well-plate. After 12 h of serum starvation to obtain cell cycle synchronization, cells were harvested at 24, 48 and 72 h of treatments of sodium mesoglycan and Prisma^®^ Skin 0.1, 0.3, 0.5 mg/mL. Equal volumes of 0.4% trypan blue stain and of the cell suspension were mixed. Approximately 10 µL of trypan blue/cell mix were put at the edge of the cover-slip of the Burker chamber and the haemocytometer grid was visualized under the optical microscope Axiovert 40 CFL (Carl Zeiss MicroImaging GmbH, Göttingen, Germany, 10×). To calculate the viable cells/mL, the average number of cells in one large square has been multiplied by the dilution factor (2) and then by 10^4^.

### 4.5. Apoptosis Detection

The percentage of the apoptotic cells was evaluated by propidium iodide (PI) (Sigma-Aldrich, St. Louis, MO, USA) staining and flow cytometry at 24, 48 or 72 h from the administration of sodium mesoglycan and Prisma^®^ Skin. The analysis was performed with FACScan cytometer (Becton-Dickinson, Franklin Lakes, NJ, USA) by Cell Quest program, version 6.0. 

### 4.6. In Vitro Wound Healing Assay

The wound healing assay was performed as reported in [54]. Briefly, HUVEC cells were seeded in a 12-well plastic plate at 3 × 10^5^ per well. After 24 h incubation, at 100% of cell confluency, a wound was produced. After removing incubation medium and washing with PBS, cell cultures were incubated in the presence of sodium mesoglycan 0.3 mg/mL, Prisma^®^ Skin 0.3 mg/mL or in growth medium as control. The wounded cells were then incubated at 37 °C in a humidified and equilibrated (5% *v*/*v* CO_2_) incubation chamber of an Integrated Live Cell Workstation Leica AF-6000 LX (Leica Microsystems, Wetzlar, Germany). A 10× phase contrast objective was used to record cell movements with a frequency of acquisition of 10 min on at least 10 different positions for each experimental condition. The migration rate of individual cells was determined by measuring the distances covered from the initial time to the selected time-points (bar of distance tool, LAS-AF software, version Lite 2.3.5, Leica microsystem CMS Gmvh). For each wound, 10 different positions were registered, and for each position 10 different cells were randomly selected to measure the migration distances. 

### 4.7. Invasion Assay

Cell invasiveness was analyzed as reported in [54]. Briefly, the upper front of trans-well Cell Culture (12 mm diameter, 8.0-fim pore size; Corning Incorporated, Corning, NY, USA) was coated Matrigel (Becton Dickinson Labware, Warszawa, Poland) diluted with 3 volumes of medium serum-free and stored at 37 °C until its gelation. Cells were plated in 350 µL of medium serum-free at a number of 9 × 10^4^/insert in the upper chamber of the trans-well. Then, 1.4 mL of supplemented EGM-2 with or without sodium mesoglycan or Prisma^®^ Skin were put in the lower chamber and the trans-well was left for 24 h at 37 °C in 5% CO_2_—95% air humidified atmosphere. After that, the medium was aspirated, the filters were washed twice with PBS 1× and fixed with 4% *p*-formaldehyde for 10 min, then with 100% methanol for 20 min. The filters so fixed, were stained with 0.5% crystal violet prepared from stock crystal violet (powder, Merck Chemicals, Darmstadt, Germany) by distilled water and 20% methanol for 15 min. After that, the filters were washed again in PBS 1× and cleaned with a cotton bud. The number of migrated cells to the lower surface was counted in twelve random fields using EVOS light microscope (10×) (Life technologies Corporation, Carlsbad, CA, USA).

### 4.8. Flow Cytometry

The analysis of CD44 protein expression was performed as reported in [55]. Briefly, HUVEC cells were harvested at a number of 1 × 10^6^ pellets were incubated in the dark on ice for 30 min in 100 µL of PBS containing APC-conjugated CD44 anti-human antibody (mouse monoclonal; BD Pharmigen, Franklin Lakes, NJ, USA), APC-conjugated human IgG1 (BD Pharmigen, Franklin Lakes, NJ, USA) was used as scrambled. The cells were analyzed with Becton Dickinson FACScan flow cytometer using the Cells Quest program, version 6.0.

### 4.9. Confocal Microscopy

After the specific time of incubation, HUVEC cells were fixed in p-formaldehyde (4% *v*/*v* with PBS) for 5 min, permeabilized in Triton ×-100 (0.5% *v*/*v* in PBS) for 5 minutes, and then incubated in goat serum (20% *v*/*v* PBS) for 30 min. Then cells were incubated with anti-ezrin (mouse monoclonal, 1:100; Santa Cruz Biotechnologies, Dallas, TX, USA), anti-moesin (mouse monoclonal 1:100; Santa Cruz Biotechnologies, Dallas, TX, USA), anti-αSMA (rabbit polyclonal, 1:100; Cusabio Life Science, College Park, MD, USA), anti-N-cadherin (rabbit polyclonal, 1:100; GeneTex, Irvine, CA, USA), anti-Twist 1/2 (rabbit polyclonal, 1:100; GeneTex, Irvine, CA, USA) anti-FAPα (rabbit polyclonal, 1:250; Santa Cruz Biotechnologies, Dallas, TX, USA), anti-fibronectin (mouse monoclonal, 1:100; Abcam, Cambrige, UK), anti-p65 (rabbit polyclonal, 1:600; Santa Cruz Biotechnologies, Dallas, TX, USA) overnight at 4 °C. After two washing steps, cells were incubated with anti-rabbit and/or anti-mouse AlexaFluor (488 and/or 555; 1:1000; Molecular Probes, Eugene, OR, USA) for 2 h at room temperature. To detect nucleus, samples were excited with a 458 nm Ar laser. A 488 nm Ar or a 555 nm He-Ne laser was used to detect emission signals from target stains. Samples were vertically scanned from the bottom of the coverslip with a total depth of 5 µm and a 63× (1.40 NA) Plan-Apochromat oil-immersion objective. Images and scale bars were generated with Zeiss ZEN Confocal Software (Carl Zeiss MicroImaging GmbH, LSM 510 Meta microscope, version 4.0 SP2).

### 4.10. Tube Formation Assay

A 24-well plate was coated with Matrigel (Becton Dickinson Labware, Franklin Lakes, NJ, USA) mixed to EGM-2 1:1 on ice and incubated at 37 °C for 30 min to allow gelation to occur. HUVEC cells were added to the top of the gel at a density of 2 × 10^4^ cells/well in the presence or not of sodium mesoglycan and Prisma^®^ Skin. Cells were incubated at 37 °C with 5% CO_2_. After 12 h, pictures were captured using EVOS light microscope (10×) (Life technologies Corporation, Carlsbad, CA, USA). The length of each tube was measured and the number of branches was calculated using ImageJ (NIH, Bethesda, MD, USA) (Angiogenesis Analyzer for ImageJ) software.

### 4.11. Sprouting Assay from Aortic Section

Thoracic aortas were dissected from isofluoran euthanized 9–12 week-old C57BL6 mice. The fibroadipose tissue was removed and the aortas were serially cross-sectioned into 1–2 mm fragments. These tissue sections were seeded on a 12-wells plate (2 pieces for well) coated with Matrigel (Becton Dickinson Labware, Franklin Lakes, NJ, USA) in EBM-2 medium. After 48 h growth medium has been replaced by treatments with sodium mesoglycan and Prisma^®^ Skin 0.3 mg/mL. After 10 days, photographs were taken at EVOS light microscope (4× and 10×) (Life technologies Corporation, Carlsbad, CA, USA). 

### 4.12. Determination of NO (Nitric Oxide) Release

To detect NO release, evaluated as (NO_2_^−^), HUVEC and SC cells (1 × 10^4^) were seeded on 96-well plates and sodium mesoglycan or Prisma^®^ Skin 0.3 mg/mL were administrated for 24 h in presence or not of LPS (lipopolysaccharide) 10 µg/mL (Sigma Aldrich). N^ω^-Nitro-l-Arginine Methyl Ester hydrochloride (L-NAME) 100 µM (Abcam, Cambrige, UK) was used as technical control as potent NOS inhibitor. NO generation was evaluated in the culture medium by Griess reaction. The amount of NO_2_^−^, evaluated as µM concentration, in the samples was calculated by a sodium NO_2_^−^ standard curve.

### 4.13. ELISA for Tumor Necrosis Factor (TNF)-α

After 24 h from the administration of LPS 10 µg/mL with or without sodium mesoglycan and Prisma^®^ Skin 0.3 mg/mL, SC supernatants were collected and secreted TNFα amount was quantified using Human TNFα ELISA kit (Elabscience Biotechnology, Hubei, China).

### 4.14. Statistical Analysis

All results are the mean ± SEM (Standard Error of Mean) of at least 3 experiments performed in triplicate. Statistical comparisons between groups were made using Student’s *t*-test, assuming a two-tailed distribution and unequal variance. Differences were considered significant if *p* < 0.05, *p* < 0.01 and *p* < 0.001.

## 5. Conclusions

In conclusion, these data could represent a significant progress in the identification of skin wound healing treatment by the use of Prisma^®^ Skin. Actually, this device promotes the new blood vessels formation and an anti-inflammatory response. Moreover, Prisma^®^ Skin and its main component, mesoglycan, are able to act in the main phases of skin wound healing, from the injury to the remodeling process [19]. Based on these results, further work is necessary to better characterize the device in the skin wound repair and the precise involvement of mesoglycan as GAGs mixture in this pathological condition.
